# Anterior Gradient 2 (AGR2) Induced Epidermal Growth Factor Receptor (EGFR) Signaling Is Essential for Murine Pancreatitis-Associated Tissue Regeneration

**DOI:** 10.1371/journal.pone.0164968

**Published:** 2016-10-20

**Authors:** Dariusz Wodziak, Aiwen Dong, Michael F. Basin, Anson W. Lowe

**Affiliations:** Department of Medicine, Stanford University, Stanford, California, United States of America; University of Szeged, HUNGARY

## Abstract

A recently published study identified *Anterior Gradient 2 (*AGR2) as a regulator of EGFR signaling by promoting receptor presentation from the endoplasmic reticulum to the cell surface. AGR2 also promotes tissue regeneration in amphibians and fish. Whether AGR2-induced EGFR signaling is essential for tissue regeneration in higher vertebrates was evaluated using a well-characterized murine model for pancreatitis. The impact of AGR2 expression and EGFR signaling on tissue regeneration was evaluated using the caerulein-induced pancreatitis mouse model. EGFR signaling and cell proliferation were examined in the context of the AGR2^-/-^ *null* mouse or with the EGFR-specific tyrosine kinase inhibitor, AG1478. In addition, the Hippo signaling coactivator YAP1 was evaluated in the context of AGR2 expression during pancreatitis. Pancreatitis-induced AGR2 expression enabled EGFR translocation to the plasma membrane, the initiation of cell signaling, and cell proliferation. EGFR signaling and tissue regeneration were partially inhibited by the tyrosine kinase inhibitor AG1478, but absent in the AGR2^-/-^ *null* mouse. AG1478-treated and AGR2^-/-^ *null* mice with pancreatitis died whereas all wild-type controls recovered. YAP1 activation was also dependent on pancreatitis-induced AGR2 expression. AGR2-induced EGFR signaling was essential for tissue regeneration and recovery from pancreatitis. The results establish tissue regeneration as a major function of AGR2-induced EGFR signaling in adult higher vertebrates. Enhanced AGR2 expression and EGFR signaling are also universally present in human pancreatic cancer, which support a linkage between tissue injury, regeneration, and cancer pathogenesis.

## Introduction

Tissue regeneration entails the reconstitution of normal architecture and function after tissue injury. The pancreas has been used for studying tissue regeneration because of well-established animal models for acute pancreatitis. In particular, the induction of acute pancreatitis with caerulein results in a highly reproducible sequence of events characterized by tissue edema, inflammation, and cell apoptosis; followed by cell proliferation and organ restoration in one week [1–4]. During this process, exocrine acinar cells undergo a metaplastic transformation characterized by dedifferentiation and the expression of developmental genes [5]. Caerulein-induced pancreatitis results in a pronounced increase in acinar cell proliferation that peaks on day 3 after the caerulein injections, and serves as a functional biomarker for tissue regeneration [5].

The Wnt and EGFR signaling pathways have both been associated with tissue regeneration during pancreatitis. Wnt signaling serves a key role in pancreatic development, and is also activated during pancreatitis [5–8]. Previous studies suggested that Wnt signaling influences acinar cell proliferation during tissue regeneration, but to what extent remained unclear [9, 10]. EGFR-mediated signaling is essential in invertebrate midgut and limb regeneration [11–13]. The role for EGFR signaling in tissue regeneration in higher vertebrates is less clear, although supportive evidence includes a reduction in pancreatitis-induced tissue damage in rats when EGF was concomitantly administered [14]. EGFR expression in acinar cells is also induced during pancreatitis [15].

A recent study established that EGFR delivery to the plasma membrane requires AGR2 expression [16]. AGR2 encodes for a thioredoxin located in the endoplasmic reticulum [17]. Similar to other endoplasmic reticulum-based thioredoxins such as protein disulfide isomerase, AGR2 forms mixed disulfides with thiol groups that may promote protein folding and assembly. Only after physically interacting with AGR2 is EGFR able to proceed to the Golgi apparatus and the remainder of the secretory pathway [16]. Without AGR2 expression, EGFR does not progress beyond the endoplasmic reticulum and cell signaling does not occur.

AGR2 is associated with tissue regeneration in fish and amphibians [18–20]. In addition, AGR2-induced EGFR signaling also results in the activation of the Hippo signaling coactivator YAP1 [16, 21], which in turn induces expression of *Amphiregulin* (AREG), an EGFR ligand [22]. Both YAP1 and AREG have also been previously associated with tissue regeneration [23, 24].

The present study employed the caerulein-induced pancreatitis model to test the hypothesis that AGR2-induced EGFR signaling is necessary for pancreatic tissue regeneration in higher vertebrates.

## Materials and Methods

### Reagents

Antibodies used in this study included: anti-E-cadherin mouse monoclonal #36 (BD Transduction); goat anti-calreticulin polyclonal antisera (Santa Cruz SC7431); rabbit anti-mouse-EGFR C-terminus (used as described by Ardito et al. [15], EMD Millipore, #06–847); rabbit monoclonal anti-phospho-EGFR for immunohistochemistry (Abcam, ab40815); anti-phospho-EGFR for protein immunoblotting (Cell Signaling, #3777); anti-EGR1 (Cell Signaling, 4153); rabbit anti-AGR2 used for protein immunoblotting was generated against GST tagged full length recombinant human AGR2; goat anti-SOX9 (Santa Cruz SC-17341); rabbit anti-YAP1 (Santa Cruz, SC15407); and rabbit anti-MKI67 (Abcam, ab15580).

### Animal Studies

Experiments using animals were performed according to protocols approved by the Stanford Administrative Panel on Laboratory Animal Care.

Wild-type mice used were female C57BL/6J, 6–8 weeks of age (The Jackson Laboratory). Pancreatitis was induced with 8 hourly intraperitoneal caerulein (Sigma) injections (0.2 mL of 10 μg/mL, 2 μg/injection) for a total of 2 days as described by Jensen et al. [5]. As described by Jensen et al., the mice were not dosed by weight. The 6-week old wild-type mice used had a mean weight of 17.9 g. The model as described by Jensen et al. was used to establish the timecourse of AGR2 expression and EGFR signaling during tissue regeneration. The first day after the caerulein injections were completed was designated as “Day 1”.

AGR2^-/-^ *null* (C57BL6/NTac) knockout mice were as previously described and were studied when 3-weeks old and a mean weight of 7.4 g [16]. For experiments that involved pharmacologically inhibiting EGFR with AG1478 or used AGR2^-/-^ *null* mice, pancreatitis was induced with a shortened protocol of 8 hourly caerulein injections over 1 day. The shorter protocol was necessary because of the reduced viability with pancreatitis of the AGR2^*-/-*^ *null* mutant mice, or the 6–8 week old C57BL/6J wild-type or heterozygous AGR2^+/-^ mice treated with the EGFR inhibitor, AG1478. As controls for the 3-week old AGR2^*-/-*^ *null* mice, 3-week old wild-type female C57BL/6J mice with a mean weight of 8.0 g were used and subjected to the same conditions to establish the presence of pancreatitis, survival rates, and serum amylase levels.

Mice treated with the EGFR-specific inhibitor, AG1478 (Fisher, Inc.), received intraperitoneal injections beginning 1 day prior to the caerulein injections at a dose of 21.4 mg/kg, which was repeated the next day while the animals received the 8 hourly caerulein injections. The mice were euthanized on the third day when AG1478 and caerulein were not administered.

Heterozygous AGR2^+/-^ mice were produced using standard breeding of similar heterozygous mice. All heterozygous AGR2^+/-^ mice used were 6–8 weeks of age and a mean weight of 16.4 g. Pancreatitis was induced using the same protocol as wild-type mice, which consisted of 8 hourly intraperitoneal caerulein injections (0.2 mL of 10 μg/mL, 2 μg/injection) per day for a total of 2 days. Similar to wild-type mice, heterozygous AGR2^+/-^ mice treated with the tyrosine kinase inhibitor AG1478 received only 1 day of 8 hourly caerulein injections because of the resultant reduced viability. Analysis of the EGR1 and MKI67 response to pancreatitis was determined on Day 1.

In this study, mice of different ages were used for the different strains of mice. All experiments performed using wild-type C57BL/6J and heterozygous AGR2^+/-^ mice to characterize survival, cell signaling, and cell proliferation were 6–8 weeks old, which was consistent with the protocol by Jensen et al [5]. Experiments performed with the AGR2^-/-^ *null* mice were 3 weeks old because of their reduced viability with age. Experiments that evaluated survival, pancreatitis histology, and serum amylase levels used 3-week old wild-type C57BL/6J mice as controls for the AGR2^-/-^ *null* mice. The ages of the mice used are noted in the figure legends.

### Polymerase Chain Reaction (PCR)

Real-time quantitative PCR (RT-qPCR) analysis was performed with 3 μg of RNA reverse transcribed with GoScript Reverse Transcriptase System (Promega, A5003) to generate cDNA, which was subjected to SYBR Green-based real-time PCR analysis with an iCycler iQ™ detection system (Bio-Rad). Primers used were directed against mouse transcripts for: ß-actin, forward (5'-GGCTGTATTCCCCTCCATCG-3') and reverse (5'-CCAGTTGTAACAATGCCATGT-3'); EGR1, forward (5'-GAGCCGAACAACCCTATGAGC-3') and reverse (5'-TGGGATAACTCGTCTCACC-3'); EGFR, forward (5'-CTGCCATTGAACGTACCCAGA-3') and reverse (5'-GCATCATGGGAGAGACAACA-3'); AREG, forward (5'-CGCTTATGGTGGAAACCTCTC-3') and reverse (5'-GGTCTTAGGCTCAGGCCATTA-3'); AGR2, forward (5'-CTGTTGCTTGTCTTGGATCTGT-3') and reverse (5'-GGAGCCAAAAAGGACCCAAAG-3'); SOX9, forward (5'-AGGAAGCTGGCACACCAGTA-3') and reverse (5'-TCCACGAAGGGTCTCTTCTC-3').

Values were expressed as the relative mRNA expression by assigning wild-type mice without pancreatitis a value of 1.

### Immunofluorescence Studies

Sections of formaldehyde-fixed pancreases were deparaffinized by immersion in xylene twice for 5 min and hydrated by immersing for 2 min in a series of 100, 80, and 50% ethanol, and finally in distilled H_2_O. The slides were counterstained with hematoxylin and eosin using standard methods. For immunofluorescence, antigen retrieval was performed in a pressure cooker set to 118°C. The slides were incubated in antigen unmasking solution (DAKO) for 3 min followed by equilibration at room temperature for 1 hr. The slides were then placed in 5% serum blocking solution (goat, horse, or rabbit serum as appropriate) for 30 min to block nonspecific binding of antibody to the tissue. The sections were incubated with primary antibody diluted in 2% serum overnight at 4°C. The respective secondary antibodies were used at predetermined dilutions. Immunofluorescence slides were mounted with media containing DAPI stain (Vectashield, Vector Laboratories).

Image analysis was performed using the Fiji ImageJ open-source software obtained at http://imagej.net/Fiji/Downloads [25]. Quantitation of nuclear EGR1, YAP1, and SOX9 utilized the binary counting methods in the image analysis software. The total number of DAPI-stained nuclei were determined in a similar manner. Determination of the number of proliferating acinar cells was achieved using nuclear MKI67 as a marker. Composite DAPI and MKI67 stained images were used to black out non-acinar cells. The image was then split into one containing the DAPI channel and another containing the MKI67 channel, followed by quantification of each channel. The DAPI positive cells were counted as described using the binary method. The number of MKI67 positive cells was determined using the ImageJ cell counter plugin. Additional details concerning the counting can be found at https://digital.bsd.uchicago.edu/docs/cell_counting_automated_and_manual.pdf. Determination of immunofluorescence co-localization was achieved using the Fiji Colo2 plugin to determine the Manders Overlap Coefficient between fluorescent images.

### Protein Immunoblotting

Pancreases on Day 1 after the caerulein injections were harvested and homogenized with a Potter-Elvehjem homogenizer in cold buffer (10 ml/g) containing 300 mM sucrose and .25 mg/ml soybean trypsin inhibitor. Protein immunoblotting was performed with 10 μg of homogenate per lane.

### Statistics

Statistical analyses were performed using GraphPad Prism software (GraphPad Software, Inc.). Results are depicted as the mean ± standard error of the mean, if not stated otherwise. For a two-group comparison, a Student's t-test was applied if the pretest for normality (D'Agostino-Pearson normality test) was not rejected at the 0.05 significance level; otherwise, a Mann-Whitney U test for nonparametric data was used. For a comparison of more than two groups, an ANOVA test, followed by a Dunnett's multiple comparisons, was applied. P values of <0.05 indicate statistical significance. No statistical method was used to predetermine sample size.

## Results

### Caerulein-Induced Pancreatitis Activates EGFR-Mediated Cell Signaling

**Fig 1:**
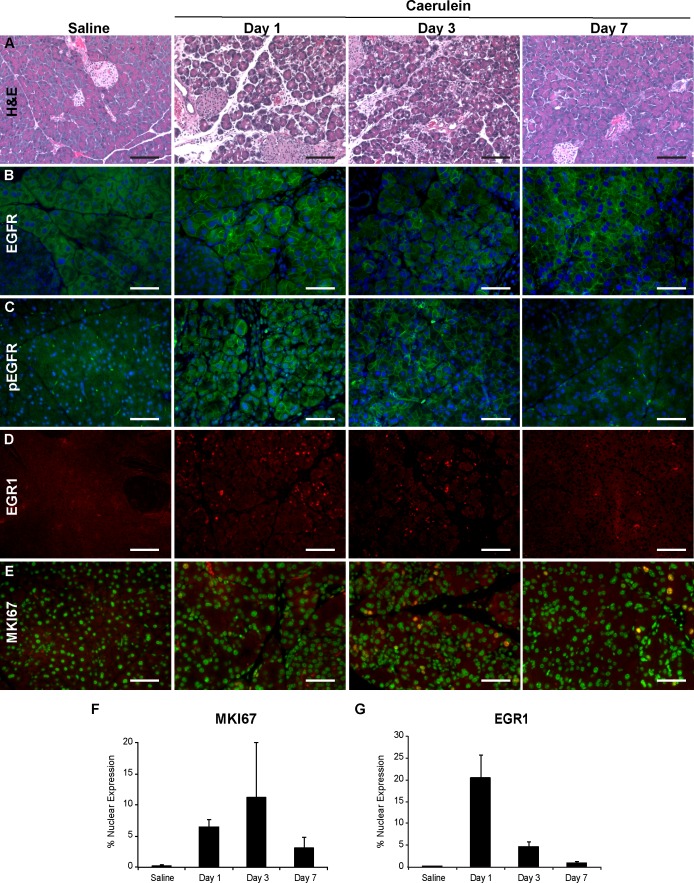
Time course of EGFR signaling and tissue regeneration in caerulein-induced pancreatitis. Pancreatitis and tissue regeneration was induced in 6–8 week old C57BL/6J mice with 2-days of 8 hourly caerulein injections. Two mice were harvested at each timepoint and processed for histology and immunohistochemistry. Day 1 represents the first day after completion of the caerulein injections. (A) hematoxylin and eosin stains of pancreases. (B) immunohistochemistry of total EGFR with an anti-cytoplasmic domain EGFR antibody (green). (C) immunohistochemistry with anti-phosphorylated EGFR antibodies (green). (D) immunohistochemistry of anti-EGR1. Nuclear EGR1 represents the activated form (red). (E) immunohistochemistry of MKI67 (red). The DAPI-stained nuclei were pseudocolored green to assist in detecting MKI67 nuclear localization, which becomes yellow. (B-D) nuclei were stained blue with DAPI. The scale bar represents 100 μm. (F) the percentage of acinar cell nuclei positive for MKI67. (G) the percentage of acinar cell nuclei positive for EGR1. The error bars represent the standard error of the mean.

The protocol for pancreatitis-induced tissue regeneration in mice as described by Jensen et al. was employed to evaluate whether EGFR cell signaling was activated [5]. Wild-type C57BL/6J mice received 8-hourly intraperitoneal caerulein injections for 2 days that resulted in pancreatitis characterized by tissue edema and infiltration by inflammatory cells (Fig 1A). Acinar tubular complexes as previously described were also detected. Also consistent with Jensen et al. was an increase in acinar cell proliferation as determined by nuclear MKI67, which served as a biomarker for tissue regeneration and peaked on Day 3 after the caerulein injections (Fig 1E and 1F) [5, 26, 27].

Pancreases were assessed with immunohistochemistry, which enabled an assessment of the extent and location of EGFR-mediated cell signaling. Anti-EGFR antibodies revealed that cell surface EGFR on acinar cells was most prominent on Day 1 and decreased over the next 2 days (Fig 1B). The activated receptor was visualized using anti-phosphorylated EGFR antibodies, which expressed a pattern similar to that of total cell surface EGFR and was most prominent the first 3 days, but absent in the saline-injected control mice (Fig 1C). EGFR signaling was also monitored through the expression and nuclear localization of the transcription factor *Early Growth Response 1* (EGR1), an immediate-early gene product induced by EGFR signaling [16, 28, 29]. Nuclear EGR1 peaked on Day 1 and declined thereafter (Fig 1D and 1G). EGFR signaling as represented by nuclear EGR1 was detected only in acinar cells (S1 Fig).

Wild-type saline-injected controls exhibited no cell surface phosphorylated EGFR, nuclear MKI67, or nuclear EGR1. Pancreatitis therefore activated EGFR signaling, which was associated with tissue regeneration as represented by cell proliferation.

### Pancreatitis Induces AGR2 Expression, EGFR Delivery to the Cell Surface, and EGFR Phosphorylation

Recently published work established that the physical interaction between EGFR and AGR2 within the endoplasmic reticulum is necessary before the receptor can progress to the Golgi apparatus and plasma membrane [16]. In human cancer cell lines, EGFR signaling is not possible without AGR2’s presence. Considering that AGR2 was previously associated with tissue regeneration in several lower vertebrates [18–20] and its importance in EGFR-mediated cell signaling, experiments were conducted to evaluate its role in the murine model of pancreatitis-associated tissue regeneration. As will be described in detail below, the following experiments were performed after only 1-day of 8 hourly caerulein injections because pancreatitis reduced the viability of mice that were AGR2^-/-^*null* mutants or pharmacologically manipulated.

**Fig 2:**
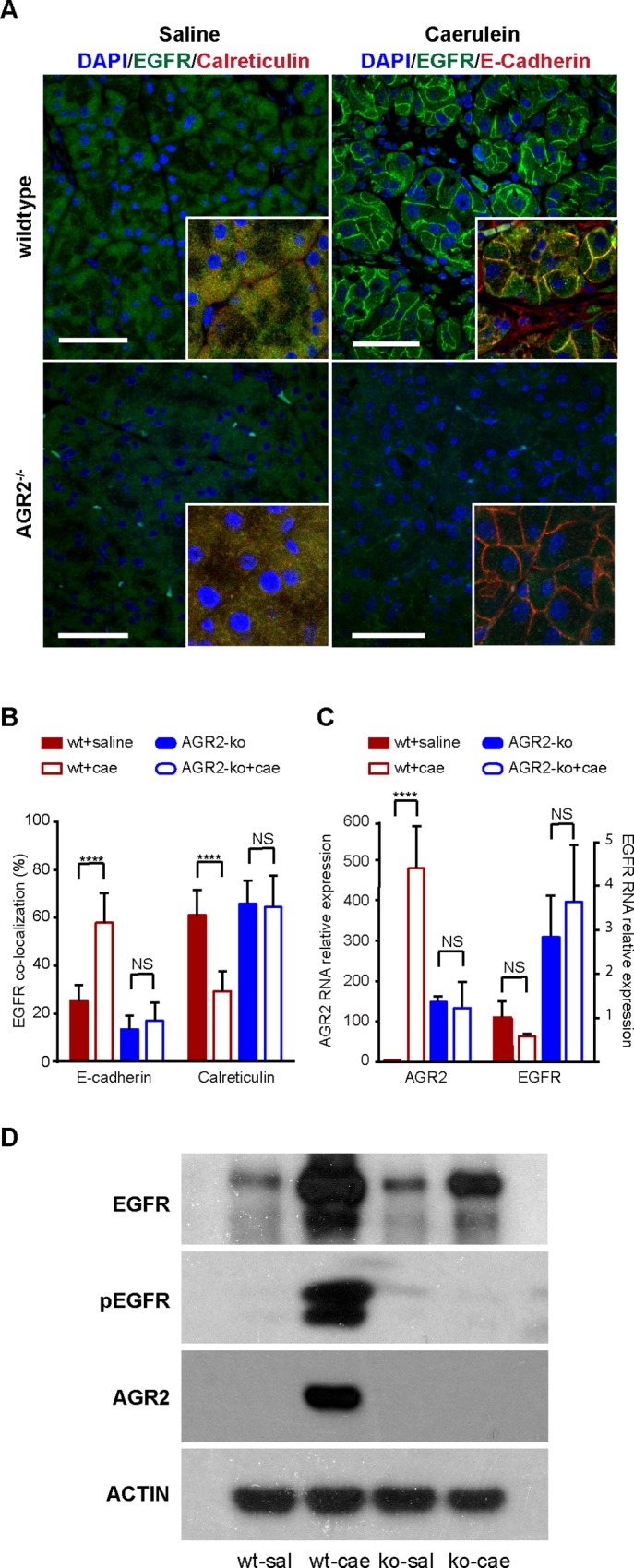
Pancreatitis induces AGR2 expression and EGFR translocation to the cell surface. (A-D) pancreatitis was induced with the 1-day protocol of 8 hourly caerulein injections in 6–8 week old wild-type (wt) and 3-week old AGR2^-/-^ (ko) mice. (A) total EGFR (green) subcellular localization with and without caerulein-induced pancreatitis as determined by immunohistochemistry and confocal imaging. The nuclei were identified with DAPI stain (blue). Scale bars = 50 μm. (A-B) calreticulin and E-cadherin served as markers for the endoplasmic reticulum and plasma membrane, respectively, and were used to quantify EGFR subcellular location. (B) Columns for wild-type (wt) represent the mean of 10 animals that each contributed 10 images. For the AGR2-ko, 3 animals were studied. Error bars represent the standard error of the mean. (C) AGR2 and EGFR RNA were determined by RT-qPCR. Values were normalized relative to wild-type mice injected with saline. The error bars represent 1 standard deviation. (D) protein immunoblots of pancreatic homogenates for total EGFR, phosphorylated EGFR, AGR2, and ß-actin as a loading control. wt = wild-type; sal = saline control; cae = caerulein; ko = AGR2^-/-^ *null*; NS = not statistically significant; **** = p-value < .00001.

In the absence of tissue injury, the pancreas does not express AGR2 protein (Fig 2D). EGFR protein is expressed, but the protein resides within the endoplasmic reticulum as shown by its co-localization with calreticulin, an endoplasmic reticulum-resident protein (Fig 2A and 2B). Histologic changes consistent with pancreatitis that included edema, inflammatory cell infiltration, and the formation of acinar cell tubular complexes [3] were observed 1 day after 8-hourly injections of caerulein, but as expected [5], the pathologic changes were less than the 2-day protocol (S2 Fig). AGR2 expression was induced by pancreatitis as detected by RT-qPCR and protein immunoblotting of pancreatic lysates (Fig 2C and 2D). EGFR mRNA expression did not change with the onset of pancreatitis (Fig 2C), but EGFR protein translocated from the endoplasmic reticulum to the plasma membrane in acinar cells as indicated by its enhanced co-localization with E-cadherin instead of calreticulin (Fig 2A and 2B). Protein immunoblots of pancreatic homogenates revealed pancreatitis induced AGR2 protein expression and EGFR phosphorylation (Fig 2D). In contrast, similar changes in EGFR translocation, AGR2 expression, or EGFR phosphorylation were not observed in saline-injected wild-type controls (Fig 2A and 2D). Consistent with previous work in which AGR2 expression in non-transformed cells resulted in translocation of EGFR to the cell surface and cell signaling [16], the present study demonstrated that pancreatitis induced AGR2 expression also achieved similar results.

### EGFR Signaling and Tissue Regeneration Is Absent in AGR2^-/-^ *null* Mice

**Fig 3:**
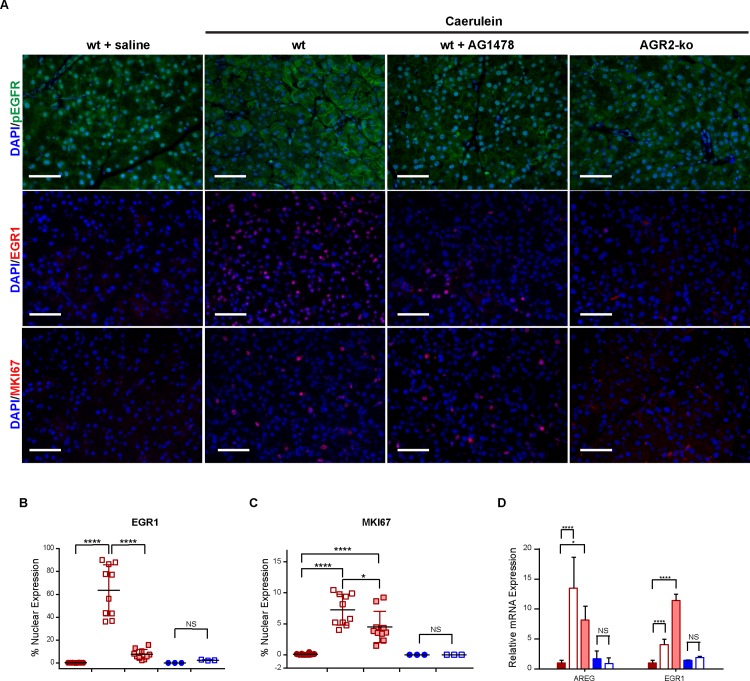
Pancreatitis-associated tissue regeneration is dependent on EGFR signaling. (A) immunohistochemistry of phosphorylated EGFR (green) and nuclear EGR1 (red) as biomarkers of cell signaling. Nuclear MKI67 (red) reflected cell proliferation. Pancreatitis was induced in wild-type (wt), wild-type pretreated with AG1478, or AGR2^-/-^ *null* (ko) mice. The wild-type mice were 6–8 weeks of age whereas the AGR2^-/-^ *null* were 3 weeks old. Pancreases were harvested 1 day after the caerulein injections. The nuclei were stained blue with DAPI. Scale bars = 50 μm. (B-C) quantitation of EGR1 (B) and MKI67 (C) nuclear localization. Each point represents the mean determination of at least ten images from each animal. Only nuclei within pancreatic acini were quantified. Closed red circles = wt + saline control; open red box = wt + caerulein; shaded red box = wt + caerulein + AG1478; filled blue circles = ko + saline control; open blue box = ko + caerulein. Black bar represents the mean. Colored error bars represent the standard error of the mean. (D) relative pancreatic mRNA expression for AREG and EGR1 as determined by RT-qPCR. Values are relative to the wt-saline controls. **** = p-value < .00001; * = p-value < .05. Error bars represent 1 standard deviation.

The impact of AGR2 expression on EGFR signaling and tissue regeneration was assessed using AGR2^*-/-*^ *null* mice. Although many EGFR^-/-^ *null* mice are embryonic lethals, survival differences are observed depending on the strain [30, 31]. Likewise, most AGR2^-/-^ *null* also exhibit a similar rate of embryonic lethality, but those that are viable at birth (3.8%) will survive for up to 6 weeks [16]. Five viable AGR2^-/-^ *null* mice were bred and studied at 3 weeks of age. The AGR2^-/-^ *null* mouse pancreases histologically appeared normal (S2 Fig). Consistent with the absence of AGR2 expression in AGR2^-/-^ *null* mice, EGFR protein remained in the endoplasmic reticulum during caerulein-induced pancreatitis (Fig 2A and 2B). All 5 AGR2^*-/-*^ *null* mice, however, were moribund within 24 hours after administration of 8 hourly caerulein injections over the course of 1 day, and either died (n = 2) or were euthanized due to agonal respirations (n = 3). Considering that AGR2^*-/-*^ *null* mice survived only 1 day after the caerulein injections, the controls consisted of 3-week old wild-type AGR2^*+/+*^ mice (n = 5) similarly treated with 8 hourly caerulein injections over 1 day. All 3-week old wild-type AGR2^+/+^ mice (n = 5) recovered from the pancreatitis induced with 1-day of 8 caerulein injections. In contrast to wild-type controls, AGR2^*-/-*^ *null* mice showed no signs of induced AGR2 expression (Fig 2C), EGFR translocation to the cell surface (Fig 2A and 2B), EGFR phosphorylation (Fig 2D), EGFR cell signaling (Fig 3A and 3B), or increase in cell proliferation (Fig 3C).

Wild-type control AGR2^+/+^ mice (n = 5) were also sacrificed on day 1 after the caerulein injections for histology. Histology of 3-week old wild-type AGR2^+/+^ and AGR2^-/-^ *null* mice with caerulein-induced pancreatitis exhibited edema and inflammatory infiltrates, but as expected to a significantly lesser degree than the 2-day caerulein injection protocol. The AGR2^-/-^ *null* mice expressed less inflammatory infiltrate and edema than the controls, a feature that is consistent with the loss of EGFR expression (S2 Fig) (see Discussion and reference [15]).

**Fig 4:**
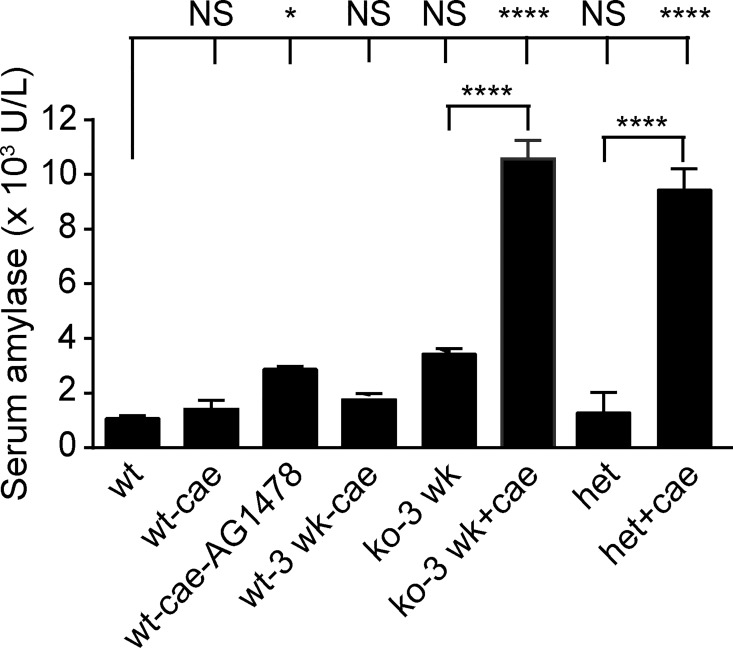
Serum amylase levels as a marker of pancreatitis severity. Serum amylase was determined on Day 1 following 1-day of 8 hourly caerulein injections. All mice were 6–8 weeks of age unless indicated. Each column represents 10 animals except for the 3-week old AGR2^-/-^ *null* (n = 3) and AGR2^+/+^ wild-type mice (n = 4). The error bar represents 1 standard deviation. **** = p-value < .00001; * = p-value < .05. wt = wild-type; cae = caerulein; 3 wk = 3 week old; ko = AGR2^-/-^ *null*; het = AGR2^-/+^ heterozygotes.

In addition to survival, serum amylase was also used as a clinical indicator for pancreatitis severity [4, 32]. AGR2^*-/-*^ *null* mice with pancreatitis exhibited serum amylase levels greater than 4-fold higher than 3-week old AGR2^+/+^ wild-type mice, and were the highest amylase levels among all groups tested (Fig 4).

### AGR2 Gene Dosage Affects Pancreatitis-Induced EGFR Signaling and Tissue Regeneration

**Fig 5:**
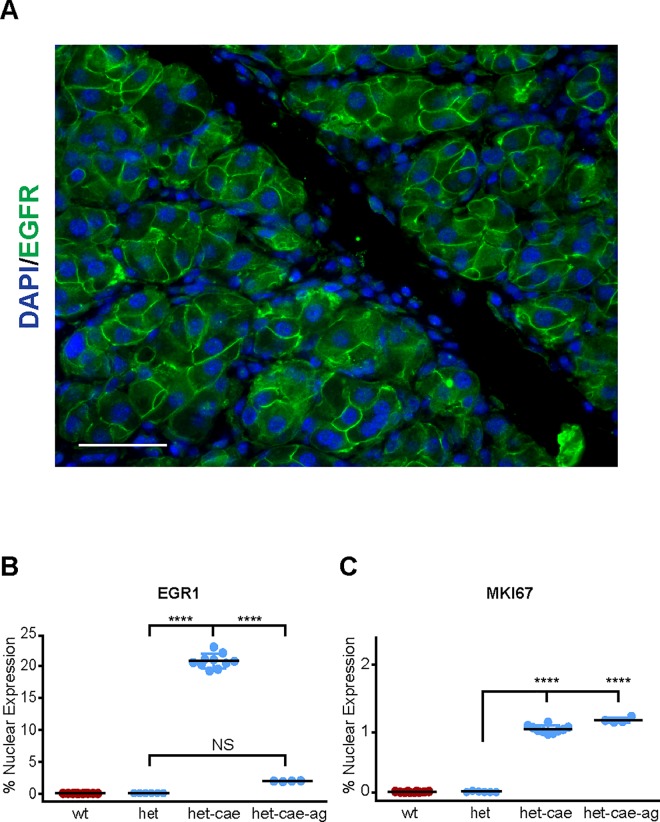
AGR2^+/-^ heterozygotes exhibit an intermediate response to pancreatitis. Immunohistochemistry performed on Day 1 of pancreatitis after 1 day of 8 hourly caerulein injections, which permitted comparisons to the AG1478-treated AGR2 heterozygotes. (A) plasma membrane EGFR (green) in an AGR2^+/-^ heterozygous mouse with caerulein-induce pancreatitis. Scale bar = 50 μm. (B-C) immunohistochemistry-determined fraction of nuclei positive for EGR1 and MKI67 in pancreatic acini. Each point represents the mean determination of at least ten images from each animal. Black bars represent the mean, and the colored error bars the standard error of the mean. **** = p-value < .00001; NS = not statistically significant. Black bar represents the mean. All mice used were 6–8 weeks old.

The effects of AGR2 gene dosage were examined using AGR2^*+/-*^ heterozygous mice. Six to 8-week old AGR2^*+/-*^ heterozygous mice with pancreatitis induced by 1 day of 8 hourly injections displayed cell surface EGFR (Fig 5A). Although EGFR signaling and cell proliferation, as represented by nuclear EGR1 and MKI67, were significantly induced in AGR2^+/-^ mice with pancreatitis on Day 1, the levels were 65% and 75% lower, respectively, than wild-type AGR2^+/+^ mice (Fig 5B and 5C). In contrast to the AGR2^*-/-*^ *null* mice, AGR2^*+/-*^ heterozygous mice could tolerate two days of caerulein injections and recovered fully from the pancreatitis after 9 days when no further cell proliferation was detected in the acinar cells (data not shown). As a biomarker of disease severity, serum amylase levels of the AGR2^+/-^ heterozygotes were markedly higher than AGR2^+/+^ wild-type mice with pancreatitis (Fig 4). AGR2^*+/-*^ heterozygotes were therefore able to survive pancreatitis, but exhibited lower levels of EGFR signaling and cell proliferation, and higher levels of serum amylase than wild-type controls. The results indicate a gene dosage effect between AGR2 and EGFR signaling that affected pancreatitis outcomes.

### Inhibition of EGFR Tyrosine Kinase Activity Reduces Cell Signaling and Tissue Regeneration with Pancreatitis

The impact of EGFR tyrosine kinase activity on pancreatitis-induced tissue regeneration was evaluated using the EGFR-specific tyrosine kinase inhibitor, AG1478 [33]. AG1478-treated wild-type C57BL/6J and heterozygous AGR2^+/-^ mice were moribund within 24 hours after 8 caerulein injections over the course of 1-day and had to be euthanized because of agonal respirations (n = 10). The severity of the pancreatitis and tissue damage after pretreatment with AG1478 was significantly greater than DMSO-treated controls as determined by serum amylase levels (Fig 4). EGFR signaling during pancreatitis was significantly reduced by AG1478 as determined by cell surface phosphorylated EGFR, nuclear EGR1, and AREG expression (Fig 3A–3D). For reasons that are not known, EGR1 mRNA expression increased with AG1478, but the levels of nuclear protein were clearly reduced (Fig 3B and 3D). Cell proliferation was induced in mice with pancreatitis that received AG1478 pretreatment, but at significantly lower levels than untreated control mice with pancreatitis (Fig 3A and 3C). Pharmacologic inhibition of the receptor's tyrosine kinase activity therefore supported a significant role for EGFR signaling in tissue regeneration and survival.

AGR2^*+/-*^ heterozygous mice pretreated with AG1478 were also moribund after 1-day of caerulein injections and were euthanized. AG1478-treated AGR2^*+/-*^ heterozygotes with pancreatitis also exhibited significantly reduced nuclear EGR1 compared to AG1478-untreated heterozygotes, but MKI67 was not significantly affected (Fig 5B and 5C).

### The Hippo Signaling Coactivator YAP1 Is Activated by Pancreatitis-Induced AGR2 Expression and EGFR Signaling

**Fig 6:**
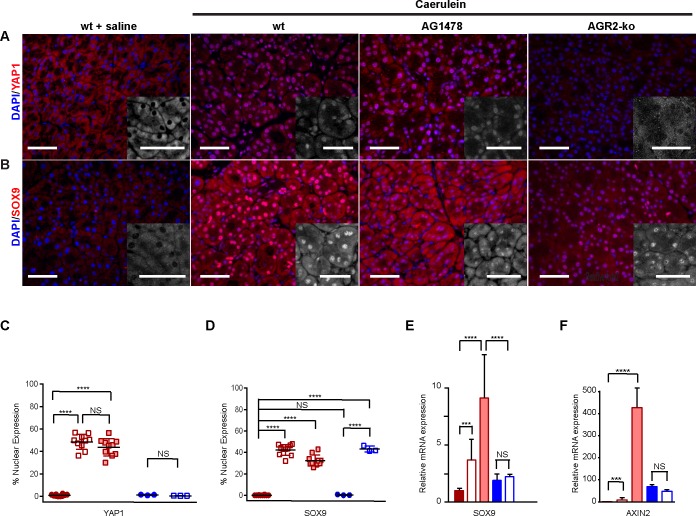
Pancreatitis-induced AGR2 expression activates YAP1, but not the progenitor-associated transcription factor, SOX9. Pancreatitis was induced with 1 day of 8 hourly injections and the mice were sacrificed on Day 1. (A-B) detection of nuclear YAP1 (A) and SOX9 (B) with and without pancreatitis in 6–8 week old wild-type mice, wild-type mice pretreated with AG1478, or 3-week old AGR2^-/-^ *null* (ko) mice. The nucleus is stained with DAPI (blue). Scale bar = 50 μm. The inserts show an enlarged view of only the YAP1 (A) and SOX9 (B) signals. Scale bar = 10 μm. (C-D) quantitation of YAP1 (C) and SOX9 (D) nuclear localization. Each point (C-D) represents the mean determination of at least ten images from one animal. Error bars represent the standard error of the mean. Closed red circles and columns = wt-saline controls; open red box and columns = wt + caerulein; filled light red box and columns = wt + caerulein + AG1478; filled blue circles and columns = ko-saline control; open blue squares and columns = ko + caerulein. (E-F) relative mRNA expression for SOX9 (E) and AXIN2 (F) as determined by RT-qPCR. Values are normalized to that of wild-type saline injected mice without pancreatitis. Error bars represent one standard deviation. NS = not statistically significant; **** = p-vale < .00001; *** = p-value < .0001.

YAP1 is a coactivator for the Hippo signaling pathway. Enhanced YAP1 activation and AREG expression have been associated with tissue regeneration in higher vertebrates [24, 34]. YAP1 activation results in its translocation from the cytosol to the nucleus where it participates in the expression of many genes, including the EGFR ligand AREG [22]. Recent studies revealed that EGFR signaling activates YAP1 through the Ajuba protein family [35]. AGR2 expression also results in YAP1 activation and induced AREG expression in human cancer cells [21]. Whether AGR2 expression also results in YAP1 activation in pancreatitis was explored. Wild-type control mice with pancreatitis exhibited a marked increase in nuclear YAP1 and AREG expression, which was not observed in similarly treated AGR2^-/-^ *null* mice (Figs 6A, 6C and 3D). EGFR inhibition with AG1478 in wild-type mice with pancreatitis still exhibited a significant increase in nuclear YAP1, which was absent in the AGR2^-/-^ *null* mice (Fig 6C). Thus, consistent with previous work [16, 21], pancreatitis-associated AGR2 expression initiates EGFR cell signaling, activates YAP1, and induces AREG expression.

### Acinar Cell SOX9 Expression Is Not Dependent on AGR2-Induced EGFR Signaling in Pancreatitis

Pancreatitis causes acinar cell metaplasia characterized by the expression of developmental genes, including features consistent with that of progenitor cells [5]. Associated with the progenitor phenotype in the pancreas is nuclear expression of the transcription factor SOX9 [36]. No nuclear SOX9 is detected in acinar cells in the normal pancreas. Consistent with previous studies [37], both AGR2^+/+^ wild-type and AGR2^-/-^ *null* mice with pancreatitis showed a marked increase in nuclear SOX9 in acinar cells (Fig 6B and 6D). AGR2 expression and EGFR signaling therefore have no effect on nuclear SOX9 that occurs in the setting of pancreatitis.

Wnt signaling is necessary for tissue regeneration in response to caerulein-induced pancreatitis in adult mice [9, 10]. Consistent with previous data [9] wild-type mice with pancreatitis led to a significant increase in AXIN2 expression, a biomarker for Wnt signaling. AGR2^-/-^ *null* mice, however, showed higher baseline AXIN2 expression, which was not induced with pancreatitis (Fig 6F). Cell proliferation was therefore absent in the AGR2^-/-^ *null* mouse despite apparent active Wnt signaling.

## Discussion

This study establishes an essential role for AGR2-induced EGFR signaling in pancreatitis-associated tissue regeneration. AGR2 is not expressed in the normal pancreas, but is induced early in the course of pancreatitis, which results in EGFR delivery to the cell surface where signaling is initiated. EGFR signaling in turn activates YAP1 and AREG expression [22, 35]. The present study therefore establishes for the first time the induction of four molecules, AGR2, EGFR, YAP1, and AREG, each of which have been individually associated with tissue regeneration in other organs, but are now all activated during pancreatitis. Recent studies have demonstrated that AGR2's residence within the endoplasmic reticulum is required to influence EGFR, YAP1, and AREG [16, 17], and that its expression represents an early initiating event. Prior evidence for the involvement of AGR2 and AREG in tissue regeneration has been restricted to vertebrates [18, 19, 24], whereas EGFR and YAP1 have been implicated in both invertebrates and vertebrates [13, 23, 38–40]. It is likely that AGR2-induced EGFR signaling and YAP1 activation is a conserved injury response for many other tissues in higher vertebrates.

Pancreatitis-induced tissue regeneration was also sensitive to EGFR tyrosine kinase inhibition as determined by cell proliferation, serum amylase levels, and survival. The effects of tyrosine kinase inhibition, however, were significantly less than the effects observed when EGFR was absence from the cell surface in the AGR2^-/-^ *null* mouse. The results support the potential significance of EGFR-mediated effects that are independent of its tyrosine kinase activity, which have also been reported by other laboratories [41–44]. For example, inhibition with the EGFR-specific tyrosine kinase inhibitor, AG1478, was much more robust for EGR1 compared to that of activated nuclear YAP1 or cell proliferation (Figs 3B, 3C and 6C). The results are consistent with previous studies demonstrating that EGFR can stimulate DNA synthesis and cell proliferation independent of its tyrosine kinase activity [44]. Previous work also established that EGFR-MAPK signaling can activate YAP1 in drosophila through its actions on the Ajuba protein family [35]. When EGFR activation of YAP1 was examined in mammalian cells, RAS-MAPK inhibition resulted in only a partial decrease in YAP1 activation, which led the authors to conclude that the process is considerably more complex in mammals compared to drosophila where regulation by RAS-MAPK activity was dominant [35]. Regardless, all EGFR-mediated effects require receptor presentation to the cell surface as shown by the absence of EGFR signaling and tissue regeneration in the AGR2^-/-^ *null* mouse. The absence of cell surface receptor would therefore affect both tyrosine kinase dependent and independent EGFR effects.

The increased severity of pancreatitis in AGR2^-/-^ *null* mice was reflected in the higher serum amylase levels and their early demise. Both AG1478-treated and AGR2^-/-^ *null* mice fared poorly, and mandated an abbreviated course of caerulein injections for subsequent analysis to be performed. Despite the poor outcomes, the histology of AGR2^-/-^ null mice with pancreatitis revealed lower levels of inflammatory infiltrate and edema than its wild-type counterpart. The use of histologic criteria [45, 46], such as the degree of inflammation, to characterize experimental pancreatitis may be misleading in the AGR2^-/-^ null mouse because EGFR signaling is required for the inflammatory response. Previous work by Ardito et al. demonstrated a significant reduction in inflammatory cells, acinar-to-ductal metaplasia, and stromal response during caerulein-induced pancreatitis when pancreatic EGFR expression was conditionally disrupted [15].

The specific cause of death was not determined other than its association with pancreatitis. All of the mice exhibited agonal breathing that required euthanasia. Severe respiratory problems are also commonly observed in severe human pancreatitis, which may also be operative in the affected mice. Although the poorer outcomes are significant and important in this study, it should be emphasized that the major focus of this study was the use of pancreatitis as a model for tissue regeneration. From this perspective, the model clearly demonstrated AGR2's importance for both EGFR signaling and cell proliferation in response to pancreatitis.

Previous studies have demonstrated a role for activated Wnt signaling in pancreatitis-induced tissue regeneration, which is consistent with the present study where AXIN2 expression was induced in response to pancreatitis. The present study is consistent with activated Wnt signaling potentially functioning upstream of AGR2 expression and EGFR signaling. The AGR2^-/-^ *null* mouse exhibited constitutive expression of AXIN2, suggesting that Wnt signaling is active, but was not induced with pancreatitis. The present study established, however, that EGFR signaling must be active for cell proliferation and tissue regeneration to occur. Disruption of EGFR signaling retarded tissue regeneration to a much greater extent than the previously published inhibition of Wnt signaling [9, 10].

Chronic tissue injury and inflammation increases the risk of developing pancreatic cancer [47, 48]. The present study mechanistically supports a strong association between tissue regeneration and pancreatic cancer. Previous work established that adenocarcinoma cell lines from the lung and esophagus are dependent on AGR2, YAP1, and AREG to maintain the transformed phenotype [21]. Likewise, inhibition of AGR2 and EGFR expression with RNA interference in pancreatic cancer cell lines reduced cell viability and the transformed phenotype [49, 50]. The present study established that the normal pancreas does not express AGR2, and EGFR signaling is inactive unless pancreatitis is induced. Similar to previous studies, tissue regeneration was initiated in pancreatic acinar cells during pancreatitis, which is also the site of EGFR signaling. Acinar cells have been proposed as the cell of origin for pancreatic cancer in animal models as they are much more susceptible to neoplastic transformation than ductal cells [51]. Enhanced AGR2 expression and activated EGFR signaling is present in all pancreatic adenocarcinomas, chronic pancreatitis, and premalignant pancreatic intraepithelial neoplasias [49, 52, 53]. Recent studies have also defined an essential role for EGFR in the early pathogenesis of pancreatic cancer. Even in the presence of an activating KRAS^G12D^ mutation, EGFR must be present for preneoplastic pancreatic lesions to develop [15, 50]. We propose that AGR2-induced EGFR signaling is a common and essential link between injury-induced tissue regeneration and the development of pancreatic cancer. AGR2 expression represents an early initiating event necessary for EGFR signaling, and thus serves as a potential target for the treatment of chronic and neoplastic pancreatic disease.

## Supporting Information

S1 Fig Nuclear EGR1 as a marker of EGFR signaling is induced only in acinar cells during caerulein-induced pancreatitis. Immunohistology of EGR1 in C57BL/6J on Day 3 after injection of either phosphate-buffered saline (top row) or 8 hourly injections of caerulein for 2 consecutive days. Tissue samples were stained for either EGR1 (red) or the nucleus with DAPI stain (blue) and presented either individually or as a composite image. Nuclear EGR1 was detected only in acinar cells in mice with pancreatitis. Scale bar = 10 μm.
(TIF)

S2 Fig Histology of caerulein-induced pancreatitis in wild-type and AGR2^-/-^ *null* mice. Hematoxylin and eosin stained tissue sections of 3-week old wild-type and AGR2^-/-^ *null* mice that had received 8 hourly intraperitoneal injections of either saline or caerulein 1 day before the tissue was harvested. Consistent with pancreatitis, caerulein treatment resulted in tissue edema, inflammatory cell infiltration, and the formation of tubular complexes (asterisk). The scale bar represents 100 μm.
(TIF)
