# Supplementary figures and images for: Anterior Gradient 2 (AGR2) Induced Epidermal Growth Factor Receptor (EGFR) Signaling Is Essential for Murine Pancreatitis-Associated Tissue Regeneration
Source: PLoS One. 2016 Oct 20;11(10):e0164968. doi: 10.1371/journal.pone.0164968 (PMC5072742; doi:10.1371/journal.pone.0164968)

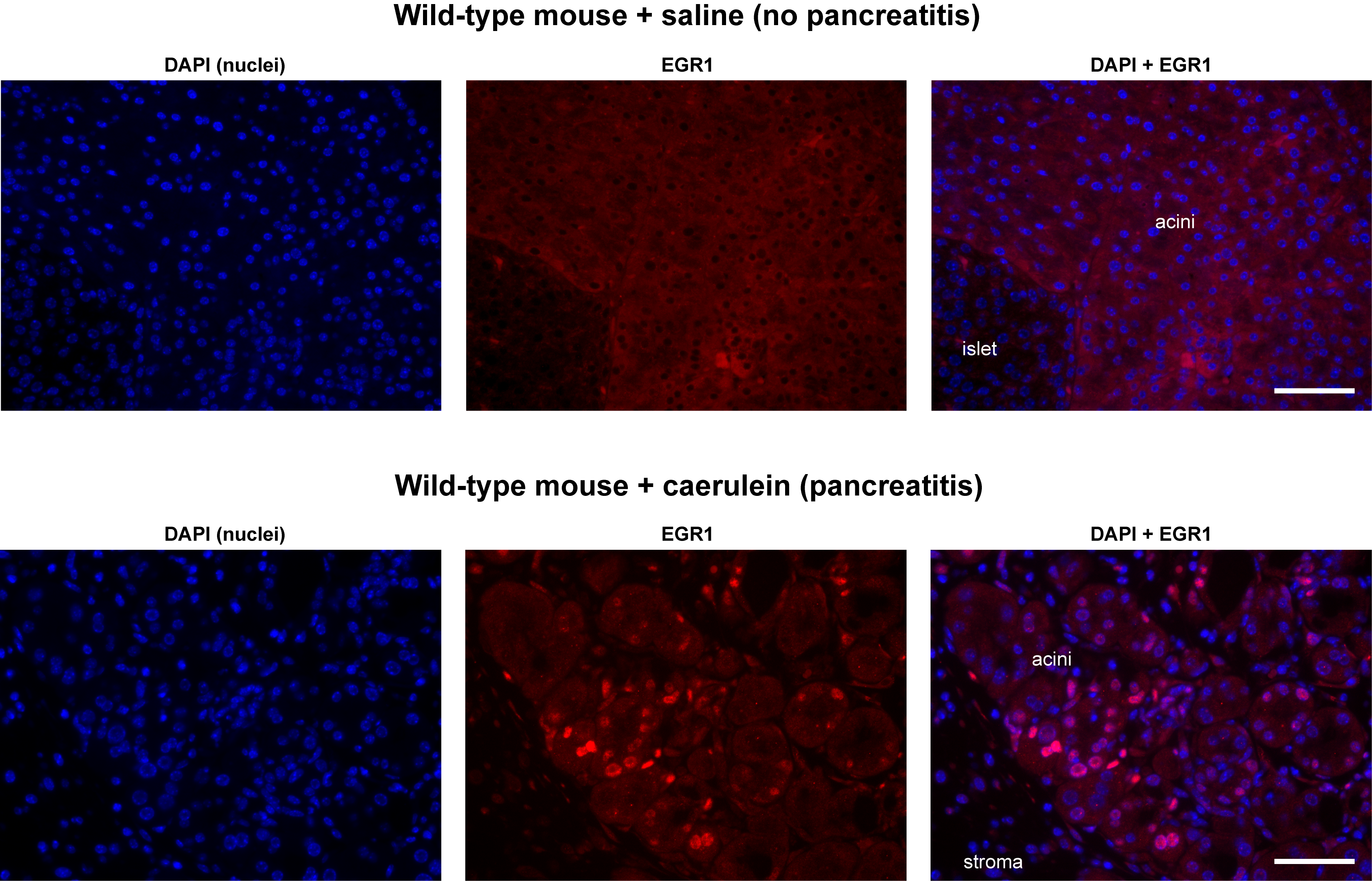

Supplement: S1 Fig — Immunohistology of EGR1 in C57BL/6J on Day 3 after injection of either phosphate-buffered saline (top row) or 8 hourly injections of caerulein for 2 consecutive days. Tissue samples were stained for either EGR1 (red) or the nucleus with DAPI stain (blue) and presented either individually or as a composite image. Nuclear EGR1 was detected only in acinar cells in mice with pancreatitis. Scale bar = 10 μm. (TIF) [file pone.0164968.s001.tif]

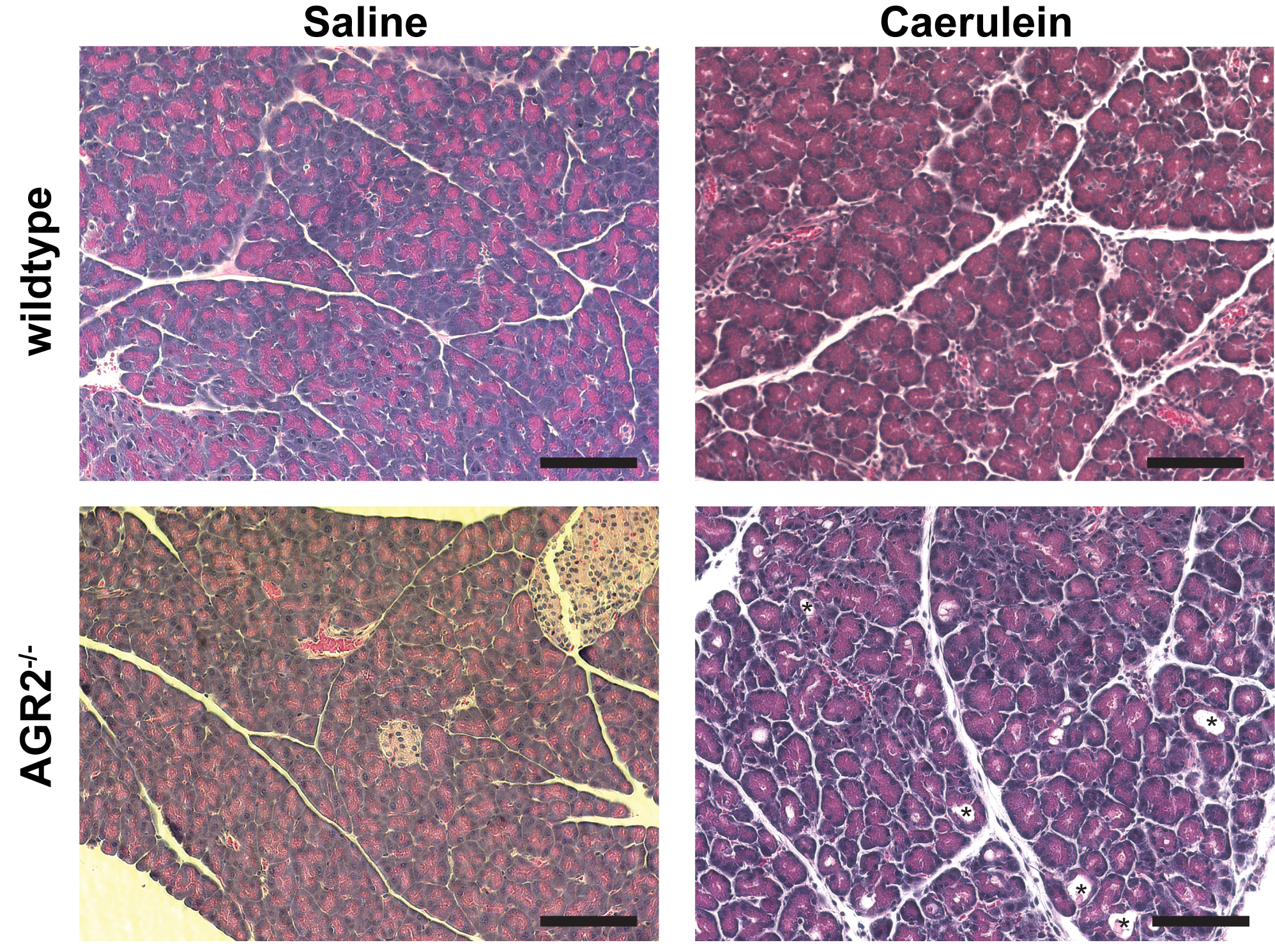

Supplement: S2 Fig — Hematoxylin and eosin stained tissue sections of 3-week old wild-type and AGR2-/- null mice that had received 8 hourly intraperitoneal injections of either saline or caerulein 1 day before the tissue was harvested. Consistent with pancreatitis, caerulein treatment resulted in tissue edema, inflammatory cell infiltration, and the formation of tubular complexes (asterisk). The scale bar represents 100 μm. (TIF) [file pone.0164968.s002.tif]
